# Dietary micronutrients intake and plasma fibrinogen levels in the general adult population

**DOI:** 10.1038/s41598-021-83217-w

**Published:** 2021-02-15

**Authors:** Alicia Padron-Monedero, Fernando Rodríguez-Artalejo, Esther Lopez-Garcia

**Affiliations:** 1 National Centre for Epidemiology, Instituto de Salud Carlos III. Madrid,, C/Monforte de Lemos 5, Madrid, Spain; 2 Department of Preventive Medicine and Public Health, School of Medicine, Universidad Autónoma de Madrid/IdiPAZ, C/Arzobispo Morcillo 2, Madrid, Spain; 3 CIBER of Epidemiology and Public Health (CIBERESP), C/Melchor Fernández Almagro 3-5, Madrid, Spain; 4 IMDEA Food Institute, CEI UAM+CSIC, Madrid, Spain

**Keywords:** Predictive markers, Risk factors, Epidemiology, Chronic inflammation, Obesity, Ageing

## Abstract

Plasma fibrinogen predicts cardiovascular and nonvascular mortality. However, there is limited population-based evidence on the association between fibrinogen levels and dietary intakes of micronutrients possibly associated with inflammation status. Data were taken from the ENRICA study, conducted with 10,808 individuals representative of the population of Spain aged ≥ 18 years. Nutrient intake (vitamin A, carotenoids, vitamin B6, vitamin C, vitamin D, vitamin E, magnesium, selenium, zinc and iron) was estimated with a validated diet history, and plasma fibrinogen was measured under appropriate quality checks. Statistical analyses were performed with linear regression and adjusted for main confounders. The geometric means of fibrinogen (g/L) across increasing quintiles of nutrient intake were 3.22, 3.22, 3.22, 3.16, and 3.19 (p-trend = 0.030) for vitamin E; 3.23, 3.22, 3.20, 3.19, and 3.19 (p-trend = 0.047) for magnesium; and 3.24, 3.22, 3.19, 3.21, and 3.19 (p-trend = 0.050) for iron. These inverse associations were more marked in participants with abdominal obesity and aged ≥ 60 years, but lost statistical significance after adjustment for other nutrients. Although dietary intakes of vitamin E, magnesium and iron were inversely associated with fibrinogen levels, clinical implications of these findings are uncertain since these results were of very small magnitude and mostly explained by intake levels of other nutrients.

## Introduction

Plasma fibrinogen is a potent marker of inflammation and coagulation^1^. This is important because inflammation is one of the mechanistic pillars of ageing^2,3^ that are shared by age-related diseases, including metabolic diseases^3^. In fact, metaflammation (metabolic inflammation accompanying metabolic diseases) is thought to be the form of chronic inflammation that is driven by nutrient excess or overnutrition, which characterizes abdominal obesity^3^, and it could be due to the activation of visceral adipose tissue macrophages^3^. Metaflammation has the same mechanism underpinning inflammaging (age-associated inflammation), which is the activation of the innate immune system in response to a variety of stimuli, including external pathogens, endogenous cell debris and misplaced molecules, nutrients and gut microbiota^3^.

Inflammation damages DNA^2,4^ and its repair^4^, and disrupts intracellular homeostasis^4^. As a result, chronic inflammatory responses predispose to most chronic disorders^2,5^, including atherosclerosis^6,7^, neoplastic transformation^4,5,8–10^, neurologic disorders^5^, diabetes^5,11^, and cardiovascular disease^5,11^. Accordingly, fibrinogen has been shown to predict cardiovascular disease incidence^1,12^ and both cardiovascular and nonvascular mortality^13^, and it is considered a potential therapeutic target^1^.

Adequate nutrition has strong anti-inflammatory effects and plays a key role in healthy aging^5^. Several plant-sourced dietary patterns, rich in fruit, vegetables and grains, as well as healthy sources of dietary fats have been inversely associated with inflammation markers^14–20^. Moreover, it has been suggested that the magnitude of the association between some nutrients and certain inflammatory markers could be stronger among populations with lifestyles and demographic factors associated with inflammation, like obesity and old age^14,21–23^; thus individuals with the latter conditions might respond better to anti-inflammatory diets^22^.

Some micronutrients found in these dietary patterns, like vitamin A, carotenoids, vitamin B6, vitamin C, vitamin D, vitamin E, magnesium, selenium, zinc and iron, could be of interest because they have anti-inflammatory properties and/or antioxidant roles^24–28^, they are co-factors of important antioxidant enzymes^29^, or they have been previously associated with fibrinogen levels^30,31^*.*

A limited number of studies have observed inverse associations between plasma fibrinogen levels and the dietary intake of vitamin E^30^, magnesium^31^, and iron^30^. By contrast, some studies did not find inverse associations between plasma fibrinogen and the dietary intake of carotenoids^32,33^, vitamin C^15,32,33^, vitamin E^32,33^, magnesium^32^, selenium^33^, zinc^32,33^, and iron^32^.

Moreover, as far as we know, no previous study has assessed the association between the dietary intake of some of the micronutrients of interest (vitamin A or vitamin B6) with plasma fibrinogen levels. There is only some information of a possible inverse association from studies using serum levels of vitamin A^22^, carotenoids^22,34,35^, vitamin B6^30^, vitamin C^15,36^, vitamin E^22,30^ and iron^30^ and plasma fibrinogen levels.

Therefore, evidence on these associations is inconclusive; also, to our knowledge, no previous population-based study that reported an inverse association with fibrinogen, has accounted for the intake of the other micronutrients or has compared the magnitude of these associations between individuals with different inflammatory status, such as younger and older adults or people with obesity and without. Thus, this study aimed to assess, in a large sample representative of the adult population of a whole country, the associations between the intake of the above micronutrients and fibrinogen levels after adjusting for main confounders, and to examine if these associations vary with age and abdominal obesity.

## Methods

### Study design and participants

Data were taken from the ENRICA study, whose methods have been reported elsewhere^37^. Briefly, ENRICA was a cross-sectional study conducted in 2008–2010 among 12,883 individuals representative of the non-institutionalized population of Spain aged 18 years and older^37^. Study participants were selected by multistage clustered random sampling. The sample was first stratified by province and by size of municipality. Clusters were then randomly selected in two stages: municipalities and census sections. Finally, the households within each section were selected by random telephone dialing. Subjects in the households were selected proportionally to the distribution of the population of Spain by sex and age^37^.

Rodriguez-Artalejo et al*.* previously reported that data collection was conducted by trained and certified staff in three stages^37^. First, a phone interview was used to obtain data on sociodemographic variables, lifestyle, health status and morbidity. In the second stage (2–4 weeks after the interview), a home visit was conducted to collect blood and urine samples and to perform a physical exam. In a subsequent home visit (third stage), food consumption was assessed with the diet history, and information was obtained on drug treatments. The mean time between the second and third stage was 10 days.

The study response rate was 51%, which is within the range of response rates in National Health Interview and Examination Surveys in Europe^38^.

All methods were carried out in accordance with relevant guidelines and regulations and all protocols of the ENRICA study were approved by the Clinical Research Ethics Committees of the University Hospital ‘La Paz’ in Madrid and the Hospital ‘Clinic’ in Barcelona. All study participants provided written informed consent.

### Study variables

#### Diet

Food consumption was obtained with the Diet History ENRICA (DH-ENRICA), a validated diet history developed from the one used in the EPIC (European Prospective Investigation into Cancer and Nutrition) Spanish cohort^39^. The DH-ENRICA is a computerized questionnaire that it is administered by a trained interviewer. Respondents are asked about food consumption during a typical week of the preceding year. The interview begins with the question: “What do you usually have to eat when you get up?” and continues asking about usual consumption on the six main intake occasions (when getting up, breakfast, mid-morning, lunch, mid-afternoon and dinner) and between those occasions, like snacking and going out for a drink. The DH-ENRICA allows to collect standardized information on 861 foods that can be cooked in 29 different ways (including mixed forms of cooking and food preservation methods). The software includes 127 sets of digitized photographs to estimate the size of food portions and provides assistance, if needed, to correctly classify some foods. The average time to complete a diet history is 45 min per participant.

The dietary intakes of the micronutrients, the macronutrients and the energy intake are estimated, by the DH-ENRICA software, from standard food composition tables^40–44^. Moreover, the DH-ENRICA software highlights weather nonrealistic values (including energy intake among other dietary measurements) were registered^39^.

In a previous validation paper from our research group^39^, micronutrient intakes from the diet history were compared against the mean of seven 24-h recalls during one year (as a gold standard), and the correlation coefficients were 0.43 for carotenoids, 0.50 for vitamin B6, 0.52 for vitamin E, 0.66 for vitamin C, 0.46 for magnesium, and 0.49 for iron^39^.

#### Fibrinogen

Plasma fibrinogen concentration (g/L) was determined in a 12-h fasting blood sample by the coagulation method. The Centre of Biological Diagnosis (CBD) of the Hospital *Clinic* in Barcelona performed the determination with appropriate quality controls.

#### Other variables

We obtained information on potential confounders that have been associated with plasma and dietary micronutrient levels^15,45^ and/or plasma fibrinogen levels^1,12,13,30,46^. These variables included age, sex, educational level (primary, secondary and university studies), smoking (never, former, and current-smoker), and physical activity at leisure time (metabolic equivalent hours/week) as assessed with the validated EPIC-Spain questionnaire^47^. We also obtained alcohol intake with the diet history, which collected data of 34 types of alcoholic beverages. Total alcohol intake was expressed in g/d; men with an average intake ≥ 40 g/d, and women with ≥ 24 g/d, were classified as heavy drinkers^48^. Weight, height and waist circumference were measured under standardized conditions. Body mass index (BMI) was calculated as weight (kg) divided by square height (m), and obesity was defined as BMI ≥ 30 kg/m^2^. Abdominal obesity was defined as waist circumference > 102 cm in men and > 88 cm in women^49^. Blood pressure was measured under standardized conditions^37^. Hypertension was defined as systolic blood pressure ≥ 140 mmHg, diastolic blood pressure ≥ 90 mmHg or the use of antihypertensive treatment. Serum glucose (mg/dL) was determined by the glucose oxidase method. Diabetes was defined as fasting serum glucose ≥ 126 mg/dL, or being treated with insulin or other hypoglycemic agents. Hypercholesterolemia was defined as serum total cholesterol ≥ 200 mg/dL or receiving lipid-lowering treatment. Participants reported the following physician-diagnosed diseases: chronic lung disease (asthma or bronchitis), cardiovascular disease (ischemic heart disease, congestive heart failure or stroke), cancer, depression and rheumatoid arthritis. Also, we asked participants about the routine use of non-steroidal anti-inflammatory medications, corticosteroids, immunomodulatory drugs, and beta interferon (because they can reduce inflammation markers), diuretics (because they can increase micronutrients excretion) and vitamin supplements. Information was checked against drug packages held at the participants´ homes.

Finally, blood level of high-sensitivity C-reactive protein (CRP) (mg/L) was measured by latex-enhanced nephelometry in the CBD in Barcelona.

### Statistical analysis

Of the initial sample comprising 12,883 individuals, we excluded 758 because they used vitamin supplements and 1317 because they had missing or implausible data on any of the study variables. Thus, the analytical sample included 10,808 individuals, who were similar to the total study sample regarding socio-demographic, lifestyle and health status variables.

To examine the study associations, we used linear regression models with fibrinogen as the dependent variable, and each of the following micronutrients intake (in quintiles) as the main independent variable: vitamin A, carotenoids, vitamin B6, vitamin C, vitamin D, vitamin E, magnesium, selenium, zinc and iron. Micronutrients were first adjusted for total energy intake using the residuals method^50^. Next, participants were categorized into quintiles of each nutrient intake and quintile 1 (lowest) was used as the reference. Given that the fibrinogen levels did not follow a normal distribution, we used the log-transformed concentration; thus, we presented the geometric mean and its 95% confidence interval (CI) of plasma fibrinogen (g/L) across quintiles of the micronutrients dietary intake. We retained for further analyses those nutrients that in the basic linear regression model adjusted for age and sex (model 1) showed an association with the log-transformed fibrinogen (p < 0.10): carotenoids, vitamin B6, vitamin C, vitamin E, magnesium and iron. We then built three multivariate linear regression models with sequential adjustment for potential confounders: Model 1 adjusted for age and sex; model 2 additionally adjusted for educational level, smoking, heavy drinking, leisure-time physical activity, energy intake, obesity, chronic diseases (chronic lung disease, cardiovascular disease, cancer, depression, hypercholesterolemia, diabetes, hypertension and rheumatoid arthritis); and model 3 further adjusted for the rest of micronutrients and the following macronutrients (quintiles of intake after adjustment for total energy intake): fibre, carbohydrates, unsaturated fats and sodium. To test the linear trend relationship between fibrinogen and each of the micronutrients of interest, we used the median value in each quintile of micronutrient intake as a continuous value in the models to obtain the P-value for linear trend of fibrinogen across the increasing quintiles of the micronutrients of interest.

We also replicated the analyses stratifying by abdominal obesity (yes/no) and age (≥ 60 and < 60 years). We tested if the results varied across strata, calculating a P-value for interaction, based on likelihood ratio tests that compared models with and without interaction terms.

Lastly, we conducted a sensitivity analysis rerunning the models after excluding participants taking anti-inflammatory medications or diuretics.

Statistical significance was set at 2-tailed p ≤ 0.05. The analyses were performed using the “Survey Data” procedures of STATA software version 15 (StataCorp LP, College Station, Texas 77845 USA), which accounted for the complex sampling design in the study.

## Results

The demographic, behavioral and clinical characteristics; macronutrients intake and blood levels of CRP, of the study participants across quintiles of fibrinogen concentration, are presented in Table 1. Compared to those in the lowest quintile of fibrinogen, participants in higher quintiles were older and less frequently men, had lower education, did less physical activity, more often had obesity and most of the diagnosed chronic diseases, had lower intake of the selected macronutrients and higher CRP levels (Table 1). Spearman correlation coefficients between the studied micronutrients intake were statistically significant in all cases and ranged from 0.27 to 0.84. Correlations between those micronutrients and plasma fibrinogen were rather small (Table 2).

**Table 1:** Characteristics of the study participants across quintiles of plasma fibrinogen in the ENRICA study (N = 10,808).

	Plasma fibrinogen (g/L)											P*
	Q 1 (lowest) (n = 2452)		Q 2 (n = 2593)		Q 3 (n = 1826)		Q 4 (n = 1832)			Q 5 (n = 2105)		
	Mean	SD	Mean	SD	Mean	SD	Mean		SD	Mean	SD	
Fibrinogen, (g/L) (mean with SD)	2.55	0.25	3.06	0.11	3.40	0.08	3.73	0.11		4.50	0.57	
Age, y (mean with SD)	39.1	14.3	44.3	15.4	47.8	15.7	51.1	16.5		54.8	17.0	< 0.001
Sex, men%	61.4		52.8		47.1		42.6			40.0		< 0.001
**Educational level, %**												
≤ Primary	18.0		23.8		29.5		35.8			40.6		< 0.001
Secondary	49.4		43.1		41.4		40.2			36.2		
University	32.7		33.1		29.2		24.0			23.3		
**Smoking status, %**												
Never smoker	47.5		46.1		46.2		47.1			48.5		0.014
Former smoker	25.3		27.9		25.4		23.4			23.7		
Current smoker	27.2		26.0		28.5		29.5			27.8		
Heavy drinker^a^, %	2.5		3.2		3.1		3.5			3.2		0.437
Leisure-time physical activity, METs-h/w (mean with SD)	33.5	24.8	30.0	22.6	27.0	20.7	24.4	18.5		23.0	18.5	< 0.001
Energy intake, kcal/d (mean with SD)	2361	652	2248	613	2188	614	2146	624		2093	630	< 0.001
**Body Mass Index**^**b**^**, %**												
Obesity	11.8		17.0		22.1		28.3			34.0		< 0.001
**Morbidity, %**												
Chronic lung disease^c^	5.2		5.6		5.4		6.0			7.5		0.010
Cardiovascular disease^d^	0.8		1.4		1.6		3.1			4.2		< 0.001
Cancer	0.5		0.5		0.6		1.3			1.9		< 0.001
Depression	4.0		5.4		6.7		8.7			9.3		< 0.001
Hypercholesterolemia	36.6		48.9		53.3		58.2			61.7		< 0.001
Diabetes	3.0		4.9		6.1		7.6			12.5		< 0.001
Hypertension	21.4		27.2		32.0		37.6			46.3		< 0.001
Rheumatoid arthritis	2.9		4.4		5.6		6.7			10.2		< 0.001
Fibre intake, (g/d) (mean with SD)	24.0	8.6	23.5	8.0	23.4	8.2	23.5	8.0		23.0	8.4	0.004
Carbohydrates intake, (g/d) (mean with SD)	244.4	77.2	232.4	72.1	226.9	72.7	224.2	72.0		221.5	73.4	< 0.001
Unsaturated fats intake, (g/d) (mean with SD)	17.1	7.9	16.2	7.6	15.5	7.5	15.2	7.3		14.6	7.4	< 0.001
Sodium intake, (mg/d) (mean with SD)	3217.3	1268.6	3056.4	1208.3	2973.3	1213.3	2994.9	1275.1		2917.9	1285.8	< 0.001
CRP, (mg/L) (mean with SD)	0.10	0.15	0.16	0.19	0.23	0.30	0.34	0.40		0.82	1.30	< 0.001

*SD* standard deviation, *MET* metabolic equivalent, *Kcal* kilocalories, *CRP* high-sensitivity C-reactive protein.

*P of the statistical significance differences between quintiles of plasma fibrinogen using the Chi-square, ANOVA or Kruskal–Wallis tests.

^a^Average alcohol intake ≥ 40 g/day in men and ≥ 24 g/day in women.

^b^Body mass index ≥ 30 kg/m^2^.

^c^Asthma or bronchitis.

^d^Ischemic heart, congestive heart failure or stroke.

**Table 2:** Spearman correlation coefficients among the micronutrients intake and fibrinogen.

	Carotenoids	Vitamin B6	Vitamin C	Vitamin E	Magnesium	Iron	Fibrinogen
Carotenoids	1.00	…	…	…	…	…	…
Vitamin B6	0.40***	1.00	…	…	…	…	…
Vitamin C	0.54***	0.43***	1.00	…	…	…	…
Vitamin E	0.41***	0.50***	0.34***	1.00	…	…	…
Magnesium	0.37***	0.76***	0.39***	0.58***	1.00	…	…
Iron	0.31***	0.76***	0.27***	0.55***	0.84***	1.00	…
Fibrinogen	0.02	− 0.08***	0.04***	− 0.06***	− 0.08***	− 0.11***	1.00

*p ≤ 0.05, **p < 0.01, ***p < 0.001.

In age- and sex-adjusted analyses (model 1), we found an inverse association between quintiles of intake of all the studied micronutrients and fibrinogen level (Table 3). When analyses were further adjusted for all potential confounders but the rest of nutrients (model 2), the dose–response associations remained statistically significant only for vitamin E, magnesium and iron. The geometric means of fibrinogen (g/L) across increasing quintiles of nutrient intake were 3.22, 3.22, 3.22, 3.16, and 3.19 (p-trend = 0.030) for vitamin E; 3.23, 3.22, 3.20, 3.19, and 3.19 (p-trend = 0.047) for magnesium; and 3.24, 3.22, 3.19, 3.21, and 3.19 (p-trend = 0.050) for iron. After additional adjustment for the rest of micronutrients and macronutrients (model 3), these associations lost statistical significance (Table 3).

**Table 3:** Geometric means of plasma concentration of fibrinogen by quintiles of micronutrient intake in the ENRICA study (N = 10,808).

	Micronutrient intake					P for trend
	Q 1 (lowest. Reference) (n = 2161) Mean (g/L) (95% CI)	Q 2 (n = 2162) Mean (g/L) (95% CI)	Q 3 (n = 2162) Mean (g/L) (95% CI)	Q 4 (n = 2162) Mean (g/L) (95% CI)	Q 5 (n = 2161) Mean (g/L) (95% CI)	
**Carotenoids**						
Range (µg/d)	< 1573	1573–2261	2261–2994	2994–4136	> 4136	
Model 1^a^	3.26 (3.22; 3.29)	3.26 (3.17; 3.34)	3.20 (3.12; 3.29)*	3.22 (3.13; 3.30)	3.20 (3.12; 3.29)*	0.011
Model 2^b^	3.22 (3.15; 3.29)	3.23 (3.12; 3.35)	3.19 (3.07; 3.31)	3.20 (3.09; 3.32)	3.20 (3.08; 3.31)	0.222
Model 3^c^	3.19 (3.10; 3.28)	3.21 (3.07; 3.35)	3.17 (3.03; 3.32)	3.19 (3.05; 3.34)	3.20 (3.05; 3.35)	0.911
**Vitamin B6**						
Range (mg/d)	< 1.6	1.6–1.8	1.8–2.0	2.0–2.4	> 2.4	
Model 1	3.26 (3.22; 3.30)	3.24 (3.16; 3.32)	3.22 (3.15; 3.31)	3.20 (3.12; 3.28)**	3.22 (3.14; 3.30)	0.043
Model 2	3.23 (3.16; 3.30)	3.21 (3.10; 3.33)	3.21 (3.10; 3.33)	3.18 (3.07; 3.30)**	3.21 (3.09; 3.33)	0.271
Model 3	3.19 (3.10; 3.28)	3.19 (3.05; 3.33)	3.20 (3.07; 3.35)	3.20 (3.05; 3.34)	3.24 (3.08; 3.40)	0.139
**Vitamin C**						
Range (mg/d)	< 64.0	64.0–90.6	90.6–118.1	118.1–161.0	> 161.0	
Model 1	3.28 (3.24; 3.32)	3.21 (3.13; 3.29)**	3.25 (3.17; 3.33)	3.18 (3.10; 3.26)***	3.22 (3.14; 3.30)**	0.004
Model 2	3.23 (3.16; 3.30)	3.19 (3.08; 3.31)	3.23 (3.12; 3.35)	3.17 (3.06; 3.29)*	3.22 (3.10; 3.33)	0.519
Model 3	3.19 (3.10; 3.28)	3.15 (3.02; 3.30)	3.20 (3.06; 3.34)	3.15 (3.01; 3.29)	3.19 (3.05; 3.35)	0.786
**Vitamin E**						
Range (mg/d)	< 7.0	7.0–8.8	8.8–10.4	10.4–12.9	> 12.9	
Model 1	3.25 (3.22; 3.29)	3.25 (3.17; 3.34)	3.24 (3.16; 3.32)	3.18 (3.10; 3.26)**	3.20 (3.12; 3.28)*	0.001
Model 2	3.22 (3.15; 3.30)	3.22 (3.11; 3.35)	3.22 (3.10; 3.34)	3.16 (3.05; 3.28)**	3.19 (3.07; 3.31)	0.030
Model 3	3.19 (3.10; 3.28)	3.20 (3.07; 3.35)	3.20 (3.06; 3.35)	3.16 (3.02; 3.31)	3.18 (3.03; 3.34)	0.524
**Magnesium**						
Range (mg/d)	< 266.2	266.2–295.0	295.0–324.6	324.6–368.4	> 368.4	
Model 1	3.27 (3.23; 3.31)	3.25 (3.17; 3.33)	3.22 (3.14; 3.30)*	3.20 (3.12; 3.29)**	3.19 (3.11; 3.28)**	0.001
Model 2	3.23 (3.16; 3.31)	3.22 (3.10; 3.33)	3.20 (3.09; 3.31)	3.19 (3.07; 3.30)*	3.19 (3.07; 3.31)	0.047
Model 3	3.19 (3.10; 3.28)	3.18 (3.04; 3.32)	3.17 (3.03; 3.32)	3.16 (3.02; 3.31)	3.17 (3.02; 3.33)	0.571
**Iron**						
Range (mg/d)	< 11.5	11.5–12.7	12.7–13.7	13.7–15.3	> 15.3	
Model 1	3.27 (3.23; 3.31)	3.24 (3.16; 3.32)	3.21 (3.13; 3.30)*	3.23 (3.14; 3.31)	3.19 (3.11; 3.28)**	0.003
Model 2	3.24 (3.16; 3.31)	3.22 (3.10; 3.34)	3.19 (3.08; 3.31)	3.21 (3.09; 3.33)	3.19 (3.07; 3.31)	0.050
Model 3	3.19 (3.10; 3.28)	3.17 (3.04; 3.31)	3.15 (3.01; 3.29)	3.16 (3.02; 3.31)	3.14 (2.99; 3.29)	0.114

*p ≤ 0.05 **p < 0.01 ***p < 0.001. P values for differences in plasma fibrinogen levels across quintiles of micronutrients intake (quintile 1 as reference) using the above linear regression models.

^a^Model 1: linear regression model adjusted for age and sex.

^b^Model 2: adjusted as model 1 and for educational level (≤ Primary, Secondary, University), smoking status (never smoker, former smoker, current smoker), heavy drinking (yes, no), leisure-time physical activity (quintiles of METs-h/w), energy intake (quintiles of kcal/d), Body Mass Index ≥ 30 (yes, no), chronic lung disease, cardiovascular disease, cancer, depression, hypercholesterolemia, diabetes, hypertension and rheumatoid arthritis (yes, no).

^c^Model 3: adjusted as model 2 and for fibre (quintiles of intake), carbohydrates (quintiles of intake), unsaturated fats (quintiles of intake), sodium (quintiles of intake) and all other micronutrients in the table (quintiles of intake).

In the stratified analyses (Table 4, model 2), the associations between vitamin E, magnesium and iron with fibrinogen levels were usually somewhat more marked among individuals with abdominal obesity and age ≥ 60 years; but in model 3 after adjustment for other nutrients, the significance of the main associations disappeared or their magnitude was extremely small (data not shown). In the sensitivity analyses with exclusion of participants taking anti-inflammatory medications and diuretics, the main results remained roughly similar, although they were even attenuated for the association between fibrinogen and iron intake in those with abdominal obesity (p for trend = 0.071) or ≥ 60 years (p for trend = 0.084).

**Table 4:** Geometric means of plasma concentration of fibrinogen by quintiles of micronutrient intake, stratified by abdominal obesity and age.

Micronutrient intake							
	Q 1 (lowest Reference) Mean (g/L) (95% CI)	Q 2 Mean (g/L) (95% CI)	Q 3 Mean (g/L) (95% CI)	Q 4 Mean (g/L) (95% CI)	Q 5 Mean (g/L) (95% CI)	P for trend	P for interaction
**Stratified by abdominal obesity**							
**Vitamin E**							
No abdominal obesity	3.18 (3.09; 3.27)	3.16 (3.01; 3.31)	3.17 (3.03; 3.32)	3.11 (2.96; 3.26)*	3.14 (2.99; 3.29)	0.086	0.323
Abdominal obesity^a^	3.56 (3.44; 3.69)	3.59 (3.39; 3.81)	3.50 (3.30; 3.71)	3.50 (3.30; 3.71)	3.51 (3.31; 3.72)	0.052	
**Magnesium**							
No abdominal obesity	3.17 (3.08; 3.26)	3.16 (3.02; 3.31)	3.14 (3.00; 3.29)	3.16 (3.02; 3.31)	3.16 (3.01; 3.31)	0.824	< 0.001
Abdominal obesity	3.60 (3.48; 3.72)	3.52 (3.32; 3.73)	3.55 (3.35; 3.75)	3.46 (3.26;3.67)**	3.48 (3.28; 3.68)**	0.002	
**Iron**							
No abdominal obesity	3.17 (3.08; 3.26)	3.17 (3.03; 3.32)	3.14 (2.99; 3.29)	3.15 (3.01; 3.30)	3.14 (3.00; 3.30)	0.275	0.023
Abdominal obesity	3.61 (3.48; 3.73)	3.53 (3.33; 3.74)	3.52 (3.32; 3.74)	3.54 (3.34; 3.75)	3.49 (3.29; 3.70)**	0.016	
**Stratified by age**							
**Vitamin E**							
Age < 60 y	3.14 (3.05; 3.22)	3.13 (3.00; 3.27)	3.12 (2.99; 3.26)	3.08 (2.96; 3.22)*	3.12 (2.99; 3.26)	0.250	0.175
Age ≥ 60 y	3.55 (3.40; 3.70)	3.54 (3.30; 3.79)	3.47 (3.24; 3.72)	3.44 (3.21; 3.70)*	3.46 (3.22; 3.70)*	0.012	
**Magnesium**							
Age < 60 y	3.14 (3.06; 3.23)	3.11 (2.99; 3.24)	3.11 (2.99; 3.24)	3.11 (2.98; 3.24)	3.13 (3.00; 3.26)	0.605	0.051
Age ≥ 60 y	3.56 (3.41; 3.71)	3.50 (3.27; 3.74)	3.52 (3.29; 3.77)	3.44 (3.20; 3.69)*	3.42 (3.19; 3.67)**	0.002	
**Iron**							
Age < 60 y	3.13 (3.05; 3.22)	3.12 (2.99; 3.26)	3.12 (2.99; 3.25)	3.12 (2.99; 3.26)	3.11 (2.98; 3.25)	0.361	0.458
Age ≥ 60 y	3.58 (3.43; 3.73)	3.51 (3.28; 3.76)	3.47 (3.23; 3.72)*	3.47 (3.24; 3.71)*	3.47 (3.23; 3.72)*	0.024	

Linear regression models adjusted as model 2 in Table 2.

Please, note that each row depicts a separate model.

^a^Waist circumference > 102 cm in men and > 88 cm in women.

*p ≤ 0.05 **p < 0.01 ***p < 0.001. P values for differences in plasma fibrinogen levels across quintiles of micronutrients intake (quintile 1 as reference) using the linear regression model.

## Discussion

Our results did not support a significant inverse dose–response association between the dietary intake of vitamin E, magnesium and iron with plasma fibrinogen concentrations after adjustment for other nutrients. In models with adjustment only for socio-demographic, behavioral and clinical characteristics, the inverse dose–response associations of dietary intake of vitamin E, magnesium and iron with plasma fibrinogen concentrations were of very small magnitude; however, these small associations were slightly more marked in older adults and participants with abdominal obesity. This could be in line with the results from a meta-analysis of randomized trials where magnesium supplementation could decrease markers of inflammation only when they were previously increased^51^, and with observations suggesting that people with obesity need higher magnesium intake to counteract their chronic low-grade inflammation^23^. However, in our study the magnitude of the studied associations, even for participants with abdominal obesity and aged ≥ 60 years, was small and even these associations were basically explained by the dietary intake of the rest of the nutrients, consequently their clinical implications are uncertain.

Our hypothesis had biological plausibility, although it was not robustly supported by the results. Specifically, vitamin E is a lipid-soluble antioxidant that preserves endothelial function^52^. Also, vitamin E increases intracellular free magnesium that protects against vascular damage caused by magnesium deficiency^53^. Moreover, vitamin E modulates the activity of enzymes and the expression of genes related to atherosclerosis^54^. Given that the endothelium has an important physiological role that regulates the coagulation and the function of platelets and monocytes^52^, the potential preservation of endothelial cell function by magnesium^27^ and antioxidants like vitamin E, among others, could stabilize inflammatory and coagulation markers^52^, including fibrinogen. As regards magnesium, it is cofactor of over 600 enzymes^55^ involved in carbohydrate, insulin^53,56^ and lipid metabolism^56^. Moreover, low levels of magnesium are associated with: systemic inflammation (reduction of antiinflammatory and increase of inflammatory cytokines)^57^, activation of the hypothalamic–pituitary–adrenal axis^58^, the metabolic syndrome^56^ and with endothelial dysfunction^59^. Finally, iron-containing enzymes participate in the hepatic detoxification of exogenous substances^24^, but when exceeding the physiological regulatory capacity, iron in this free form may act as a prooxidant agent^60^. In any case, the relation between iron dietary intake and inflammation markers is complex and it could partially be explained by its original food source (heme or non-heme)^32^.

Few studies have previously assessed the association between the dietary intake of micronutrients and fibrinogen levels. Consistently with our results, Kim et al*.*, in a 20-year follow-up of a cohort of young adults, found that magnesium intake (measured at years 0 and 7) was inversely associated with subsequent levels of fibrinogen^31^. James et al*.* in a cross-sectional study of people aged ≥ 15 years, found that those with higher fibrinogen levels had significantly lower dietary intakes of iron and vitamin E^30^. These findings are coherent with our results before adjustment for the rest of the nutrients.

By contrast, De Oliveira Otto et al*.* in a cross-sectional analysis found a direct association between dietary intake of magnesium and fibrinogen level, but the association lost significance after adjustment for the dietary intake of zinc and heme iron^32^. However, no associations were found between fibrinogen levels and the dietary intakes of iron, zinc, vitamin C, vitamin E or β-carotene. These authors suggested that the inconsistencies assessed for some nutrient-marker relationship could be explained by the complexity of diet and the inherent difficulty to identify independent nutrient associations^32^. Likewise, Wannamethee et al*.* in a cross-sectional study, reported that plasma levels, but not the dietary intake of vitamin C, had an inverse association with fibrinogen in older men^15^; and Corley et al*.* in a cross-sectional study of adults ≥ 70 years reported inverse associations of the Mediterranean dietary pattern and combined fruit and vegetable consumption with fibrinogen levels, but no association was observed for the dietary intake of vitamin C, vitamin E, β-carotene, zinc and selenium^33^; although, neither of these studies analyzed the association with magnesium or iron.

### Strengths and limitations

A main strength was the large study sample that was representative of the adult population of Spain. Also, food and nutrient intake was obtained through a validated diet history, and analyses were adjusted for many potential confounders. Our study has several limitations. First, the main limitation was the cross-sectional study design, so the temporality of the associations is unknown and causal inference is limited. Second, fibrinogen was measured only once, so the associations might be underestimated due to regression dilution bias. Moreover, if fibrinogen levels in some individuals reflected only an acute condition rather than a chronic inflammatory state, this might have also reduced the actual associations; however, this is likely to have had only a minor effect in our study because, according to the study protocol, participants with fever or other acute health problems postponed the blood draw until complete recovery. Third, simultaneous adjustment for micronutrients that share some food sources (e.g., green vegetables, whole grains and nuts are rich in vitamin E and magnesium) it is controversial, because it may represent an over-adjustment. Although the aim was to assess the independent association of each nutrient with fibrinogen, the fact was that mutual adjustment for all micronutrients led to a loss of statistical significance of the observed associations. Fourth, the fibrinogen range considered as normal in humans is 2–4 g/L, so most study participants were within the normal range. Some recent studies have found that age interacts with fibrinogen levels > 3.1 g/L to increase cognitive impairment^61^, and that fibrinogen presents a significant continuous trend with the risk of cardiovascular death^62^. Although we cannot completely rule out a benefit of reducing fibrinogen even in subjects with normal levels, the clinical significance of changes in fibrinogen within the normal range should be interpreted with caution. Fifth, we did not include in the models other nutrients like protein, total fat, vitamin A, vitamin D, selenium and zinc dietary intake as covariates. The rationale was that, although there was a theoretical ground to include most of them, they showed no association (p < 0.10) with the log-transformed fibrinogen in the basic linear regression model (model 1). Moreover, its inclusion in the adjusted models would have not provided any added value but would have decreased the parsimony of the model. Furthermore, since some of these nutrients, like vitamin A and zinc, were strongly correlated to some of the studied micronutrients, including them in the adjusted models would have produced data multicollinearity and thus, reduced the precision of the coefficient estimates and the statistical power. And sixth, despite using a validated diet history and excluding participants with an implausibly high or low energy intake level, certain misreporting and misclassification of micronutrient intake is possible. Moreover, recall of past food consumption may be distorted by current consumption, and certain social desirability bias, which tends to over-represent the recall of healthy food, may also operate. These measurement errors usually tend to lead the observed associations towards the null.

## Conclusions

The dietary intakes of Vitamin E, magnesium and iron were not inversely associated with lower fibrinogen levels after adjusting for other nutrients. However, in participants with abdominal obesity and aged ≥ 60 years the relation appeared to be slightly more prominent. Nevertheless, the magnitude of the observed associations was very small, even for older adults and participants with abdominal obesity, and virtually lost statistical significance after adjustment for other nutrients, so their clinical implications are uncertain.

## Data Availability

The datasets used and analyzed during the current study are available from the corresponding author on reasonable request.
